# Hsa_circ_0020095 Promotes Oncogenesis and Cisplatin Resistance in Colon Cancer by Sponging miR-487a-3p and Modulating SOX9

**DOI:** 10.3389/fcell.2020.604869

**Published:** 2021-01-15

**Authors:** Yanlai Sun, Zhen Cao, Junqi Shan, Yang Gao, Xin Liu, Dejian Ma, Zengjun Li

**Affiliations:** ^1^Department of Gastrointestinal Cancer Surgery, Shandong Cancer Hospital and Institute, Shandong First Medical University and Shandong Academy of Medical Sciences, Jinan, China; ^2^Shandong First Medical University and Shandong Academy of Medical Sciences, Jinan, China

**Keywords:** colon cancer, circ_0020095, miR-487a-3p, SOX9, proliferation

## Abstract

**Objectives:**

Colon cancer (CC) currently ranks as the third most common human cancer worldwide with an increasing incidence and a poor prognosis. Recently, circular RNAs have been reported to regulate the progression of diverse human cancers. However, the role of circRNA hsa_circ_0020095 in CC remains largely unclear.

**Methods:**

Expression levels of the related circRNAs, microRNAs and mRNA in CC tissues and cells were determined. The impacts of circ_0020095 or miR-487a-3p on CC cells were examined at the indicated times after transfection. Meanwhile, a luciferase-reporter experiment was employed to validate the interplay between miR-487a-3p and circ_002009695 or SOX9. Moreover, the *in vivo* tumor growth assay was applied to further evaluate the effects of circ_0020095 knockdown on CC progression.

**Results:**

We demonstrated that circ_0020095 was highly expressed in CC tissues and cells. The proliferation, migration, invasion, and cisplatin resistance of CC were suppressed by silencing circ_0020095 *in vitro* and *in vivo* or by ectopic expression of miR-487a-3p *in vitro*. Mechanistically, circ_0020095 could directly bind to miR-487a-3p and subsequently act as a miR-487a-3p sponge to modulate the activity by targeting the 3′-UTR of SOX9. Interestingly, overexpression of circ_0020095 dramatically reversed the suppressive effects of miR-487a-3p mimics on CC cells.

**Conclusion:**

Circ_0020095 functions as an oncogene to accelerate CC cell proliferation, invasion, migration and cisplatin resistance through the miR-487a-3p/SOX9 axis, which could be a promising target for CC treatment.

## Introduction

Colon cancer (CC) is known as one of the most frequent digestive cancers in the world with a high incidence and poor prognosis (Weinberg and Marshall, 2019). Despite the huge progress of therapeutic modalities, CC still ranks as the third primary cause of tumor-associated death (Banerjee et al., 2017). At present, CC incidence rises dramatically with age due to the accumulation of random somatic mutations, and more than ninety percent of CC cases occur after 50 years of age (Cappell, 2003). Multiple therapeutic options, including surgical removal, radiotherapy and chemotherapy, can be used for the treatment of CC patients. The mortality of CC has been decreased by more than 30% in recent decades (Howe et al., 2006). Nevertheless, the 5-year survival rate of CC patients at advanced stages is still only approximately 10%, which is mainly related to the multidrug resistance to chemotherapy drugs in advanced patients (Wang et al., 2014). In addition, recurrence has been observed in a substantial proportion of advanced CC patients after surgery (Gerner et al., 2018; Weinberg and Marshall, 2019). Therefore, it is essential to better understand the molecular mechanisms of CC to develop more effective approaches for the treatment of CC.

It is generally known that only approximately two percent of the human genome possesses protein-coding capacity, and most transcripts are generated as non-coding RNAs, which used to be considered transcriptional noise (Yang et al., 2019). As one of the most common subtypes, circular RNAs (circRNAs), which have a stable circular structure, were recently found to be widely expressed in mammalian cells (Qu and Adelson, 2012). Since the first report was published, circRNAs have been found to play a role in various cellular physiological processes, such as cell proliferation, apoptosis and differentiation (Holdt et al., 2018; Patop and Kadener, 2018). Dysregulation of circRNA expression has been observed in multiple human diseases, such as cancers, neurodegenerative disorders, and metabolic diseases (Haque and Harries, 2017). Recently, a number of circRNAs have been reported to contribute to the progression of CC (Ju et al., 2019). However, the pathogenesis of CC is still not completely understood. Circ_0020095 originates from the ATRNL1 gene and consists of the head-to-tail splicing of exons 9–18. It is a novel circRNA that was identified to be dysregulated in CC tissues in a microarray analysis (Zhang et al., 2019). Its functional role in CC remains undetermined.

Other studies have also elucidated that circRNAs suppress the inhibitory effects of miRNAs on target RNAs by competitively binding miRNAs (Militello et al., 2016). This competitive relationship represents a novel mechanism of gene regulation that has a major role in the physiology and development of cancer (Kulcheski et al., 2016). In our preliminary experiments, we screened and identified five potential target miRNAs and found that the level of miR-487a-3p was significantly regulated by circ_0020095 in CC cells. Therefore, we speculated that circ_0020095 may influence the CC process by regulating miR-487a-3p. MicroRNAs (miRNAs) can be widely involved in a variety of biological processes, such as cell differentiation, proliferation, apoptosis and metastasis, by translation inhibition or direct degradation of mRNAs to negatively regulate specific target genes (Hammond, 2015; Peng and Croce, 2016). Currently, an increasing number of miRNAs, such as miR-193a (Teng et al., 2017), miR-137 (Sakaguchi et al., 2016), miR-34a (Bu et al., 2016) and miR-143 (Gomes et al., 2018), have been found to be associated with the occurrence and development of CC by promoting or preventing the malignant biological behavior of CC. MiRNAs are expected to become novel markers for the diagnosis, treatment and prognosis of CC (Slattery et al., 2018). Studies have demonstrated that miR-487a-3p is a vital biomolecule regulating prostate cancer, breast cancer and other cancers (Yang et al., 2018; Wang et al., 2019). SRY-box transcription factor 9 (SOX9), as a chemoradiotherapy-sensitive gene in colorectal cancer patients, could be a suitable biomarker to predict the relapse after the treatment (Du et al., 2019). In human non-small cell lung cancer, miR-592 functions as a tumor suppressor by targeting SOX9 (Li et al., 2017). Therefore, we speculated that miR-487a-3p might also be a crucial therapeutic target for CC.

In this study, we aimed to investigate the biological functions of circ_0020095/miR-487a-3p and further elucidate the underlying molecular mechanisms in CC which may become a promising therapeutic target for CC patients.

## Materials and Methods

### Collection of CC Tissues and Cell Lines

Twenty CC tissues and their paired normal tissues were obtained from patients who received treatment at Shandong Cancer Hospital and Institute between 2013 and 2018, and written informed consent was obtained from every patient. This study was approved by the ethics committee of Shandong Cancer Hospital and Institute. A healthy human colon cell line (NCM460) and CC cell lines (HT29, SW480, SW620, and HCT116) were provided by the Type Culture Collection of the Chinese Academy of Sciences (Shanghai, China). Cells were cultured in RPMI-1640 medium (HyClone, United States) containing 10% fetal bovine serum (FBS) and kept at 37°C in a cell incubator with 5% CO2 and 95% air.

### RNA Extraction and Quantitative Real-Time PCR (RT-PCR) Assay

RNA extraction from CC tissues and cells was carried out using TRIzol reagent (Invitrogen, United States) following the manufacturer’s instructions. After the concentration of RNA was determined via a NanoDrop 2000c (Thermo Scientific, United States), 3 μg of RNA was used as template to produce cDNA using a Bestar qPCR RT kit (DBI Bioscience, China). The RT-PCR of circRNAs, miRNAs and mRNAs was completed using Bestar qPCR MasterMix (DBI Bioscience) on an ABI 7500 system (ABI Biosystems, United States). The sequences of the primers used in this study are shown in Table 1.

**TABLE 1:** All primers of qRT-PCR analysis used in the current study.

Gene	Primers	
	
	Forward (5′-3′)	Reverse (5′-3′)
GAPDH Circ_0020095 MiR-487a-3p SOX9 miR-485-3p miR-338-3p miR-656-3p miR-660-5p U6	TGTTCGTCATGGGTGTGAAC GCTTATTGGAATGCACCACA AATCATACAGGGACATC AGGAAGTCGGTGAAGAACGG CCGCTCGAGATGCGGCTT TGGG AAGC TGCGGTCCAGCATCAGTGAT ACACTCCAGCTGGGAAT ATTATA CAGTCA TACCCATTGCATAT CGGAGTTG CTCGCTTCG GCAGCACA	ATGGCATGGACTGTGGTCAT GTTTCTGGAACAAGC CAAGTG TGCGTGTCGTGGAGTC CGCCTTGAAGATGGCGTTG CGGGGTACCAAGATGCTT CTA GATGCCC CCAGTGCAGGGTCCGAGGT CTCAACTGGTGTCGTGGA GTCG GCAATTCAGTTGAGAGA GGUUG GTGCAGGGTCCGAGGT AACGCTTCACGAATTTGCGT

### RNase R Resistance Analysis

Circ_0020095 and its linear isoform were incubated at 37°C with RNase R (5 U/mg, Epicenter) for half an hour. Subsequently, the treated RNAs were reverse transcribed with the indicated primers and analyzed by qRT-PCR.

### Fluorescence *in situ* Hybridization (FISH)

Biotin-labeled specific RNA probe of circ_0020095 was obtained from RiboBio (Shanghai, China). Exponentially growing HT29 cells were collected and fixed in 4% formalin and then hybridized in hybridization buffer containing biotin-labeled circ_0020095. Signals were detected via a tyramide-conjugated Alexa 488 fluorochrome TSA kit.

### RNA Transfection

Two specific siRNAs against circ_0020095 (siRNA#1 and siRNA#2) and a negative control (siRNA-NC) and a miR-487a-3p mimics, inhibitor and miR-NC were all synthesized by RiboBio (Shanghai, China). Transfection into HT29 and SW480 cells was carried out using Lipofectamine 3000 (Invitrogen) following the manufacturer’s protocol.

### Cell Counting Kit-8 (CCK-8) Assay

Cell Counting Kit-8 (Dojindo, Rockville, MD, United States) reagent was added to HT29 and SW480 cells to determine cell proliferation. In brief, 2 × 10^4^ HT29 and SW480 cells were seeded into 96-well plates. After 24 h of culture, 10 μl CCK-8 solution was added into each well and incubated for 10 min. Subsequently, absorbance was measured at 450 nm.

### Cisplatin Treatment

Treated HT29 and SW480 cells were collected at exponential growth phase and seeded into 96-well plates, followed by incubation with cisplatin (0, 5, 10, 15, and 25 μg/ml) for the indicated times. Then, cell proliferation activity was examined by the CCK-8 assay.

### Colony Formation Assay

Treated HT29 and SW480 cells were collected at exponential growth phase and seeded into 96-well plates at a concentration of 2 × 10^4^ cells per well. After 2 weeks of culture at 37°C, visible colonies were fixed in 4% paraformaldehyde followed by staining with Giemsa solution. The number of colonies in both the control and experimental groups was counted under a microscope at 10× magnification.

### Cell Apoptosis Analysis (Flow Cytometry Analysis)

Treated HT29 and SW480 cells were collected at exponential growth phase and seeded into 96-well plates at a concentration of 2 × 10^4^ cells per well. After staining with propidium iodide and Annexin V, HT29 and SW480 cells were subjected to cell apoptosis using a flow cytometer (FACScan, United States), and the results were analyzed by CELL Quest 3.0 software.

### Wound-Healing Assay

Treated HT29 and SW480 cells were harvested and seeded into 35-mm dishes at a concentration of 1 × 10^3^ cells per well and cultured at 37°C until confluence. A straight scratch was made on the cell surface using a sterile pipette tip. Images were taken 0 and 24 h after scratching, and the width of the scratch was measured under a microscope.

### Transwell Assay

Transwell chambers coated with or without Matrigel matrix (BD Bioscience, United States) were employed to estimate the cell migration and invasion of HT29 and SW480 cells. Treated HT29 and SW480 cells were harvested and resuspended in culture medium to a final concentration of 1 × 10^5^ cells/ml. The upper chamber was filled with 200 μl of cell suspension, and the lower chamber was filled with 500 μl FBS-containing culture medium. After 24 h of incubation at 37°C, migratory and invasive CC cells were fixed with 70% ethanol and stained with crystal violet. The number of migratory and invasive cells was counted under a microscope.

### Dual Luciferase Reporter Assay

Circ_0020095 sequence containing wild-type (WT) or mutated (MUT) miR-487a-3p binding site was synthesized and inserted into the pmirGLO Dual-luciferase miRNA Target Expression Vector (Promega, WI, United States). The recombinant reporter plasmid was named circ_0020095-WT or circ_0020095-MUT. circ_0020095-WT or circ_0020095-MUT was cotransfected with miR-487a-3p or miR-NC into HT29 and SW480 cells using Lipofectamine 3000 (Invitrogen, United States). Luciferase activities of HT29 and SW480 cells were measured after 48 h of cotransfection by using the Luciferase Reporter Assay System (Promega, WI, United States). Similar to the above, a SOX9 sequence containing the WT or MUT miR-487a-3p binding site was amplified and subcloned into the pmirGLO vector to generate SOX9-WT or SOX9-MUT reporter plasmid. The transfection procedure was carried out as described above.

### *In vivo* Tumor Growth Assay

HT29 cells stably expressing circ_0020095 siRNAs or si-NC were harvested and resuspended in culture medium at a concentration of 2 × 10^5^ cells/ml. Next, 200 μl HT cell suspension was inoculated into the right flanks of nude mice (male, 8 weeks old). Animals were obtained from the University of Jinan-Shandong Academy of Medical Sciences. Animal manipulations were approved by the Institutional Animal Care and Use Committee of the Hospital. Tumor volume and weight were examined every week until 4 weeks after inoculation.

### Statistical Analysis

Data in this study are shown as the means ± standard deviation (SD) and were analyzed with SPSS (version 20.0, SPSS, Chicago, United States). The significance of the difference between the control and experimental groups was estimated via Student’s *t* test, and P less than 0.05 was considered statistically significant.

## Results

### Circ_0020095 Was Identified to Be Increased in CC

**FIGURE 1:**
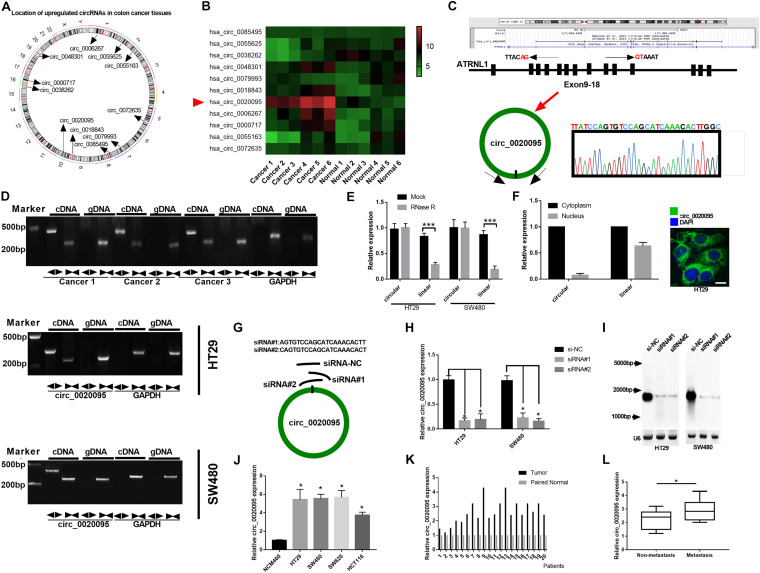
Circ_0020095 was determined to be increased in CC. **(A)** Location of upregulated circRNAs in CC tissues. **(B)** qRT-PCR analysis of 11 circRNAs in six paired CC tissues. **(C)** Schematic diagram of the genomic position and formation of circ_0020095. Sanger sequencing was used to examine the back-splice junction of circ_0020095. **(D)** Circ_0020095 was examined using semiquantitative PCR with divergent and convergent primers in cDNA and gDNA extracted from CC tissues and cell lines (HT29, SW480, and SW620). GAPDH was used as a linear RNA control. **(E)** qRT-PCR validation of circ_0020095 and linear mRNA was performed in HT29 and SW480 cells in the presence of RNase R. **(F)** The distribution of circ_0020095 in HT29 cells was detected using FISH, and DAPI was used to stain the nucleus. **(G)** Two siRNAs (siRNA#1 and siRNA#2) were designed to target the back-splice junction of circ_0020095, and siRNA-NC (si-NC) was used as a negative control. **(H,I)** Circ_0020095 expression in HT29 and SW480 cells transfected with si-NC, siRNA#1, and siRNA#2 was measured with Northern blots. **(J)** Expression level of circ_0020095 in four CC cell lines, HT29, SW480, SW620, and HCT116. NCM460 was used as the control group. **(K)** Circ_0020095 expression in 20 pairs of CC and normal tissues was tested by qRT-PCR. **(L)** Circ_0020095 expression in metastatic and non-metastatic CC tissues. ◀▶ : divergent primers ▶◀ : convergent primers. *P < 0.05, **P < 0.01, ***P < 0.001.

A previous study identified 334 dysregulated circRNAs in CC tissues by microarray analysis; and we focus on the top 11 upregulated circular RNAs that may involve in the carcinogenesis of CC (Zhang et al., 2019). Figure 1A indicates their genome location. In our cohort, we randomly selected 6 paired CC tumor tissues and adjacent normal tissues and tested the expression of these 11 circular RNAs, and we found that circ_0020095 was the most dysregulated circRNA between the CC tumor tissues and paired normal tissues (Figure 1B). Circ_0020095 originates from the ATRNL1 gene and consists of the head-to-tail splicing of exons 9–18, and Sanger sequencing was used to validate its circular structure (Figure 1C). Convergent and divergent primers were designed to amplify linear and circular RNA isoforms based on templates of cDNA and gDNA extracted from three CC tissues and HT29 and SW480 cell lines using semiquantitative PCR. The results indicated that convergent primers could only amplify linear RNAs (the band close to 200 bp) in the cDNA and gDNA groups while circ_0020095 (the band close to 500 bp) could only be produced in the cDNA group but not in the gDNA group by divergent primers (Figure 1D). Moreover, in HT29 and SW480 cells, we found that the circular isoform was resistant to RNase R treatment, while the linear isoform was clearly digested by RNase R (Figure 1E). In addition, the FISH assay indicated that circ_0020095 was predominantly located in the cytoplasm, while its isoform was located in both the cytoplasm and nucleus (Figure 1F). Two siRNAs (siRNA#1 and siRNA#2) against circ_0020095 were synthesized to silence the expression of circ_0020095 in HT29 and SW480 cells (Figure 1G). Northern blots analysis of circ_0020095 indicated that siRNA#1 and siRNA#2 transfection resulted in a significant downregulation of circ_0020095 in HT29 and SW480 cells (Figures 1H,I). Subsequently, we found that circ_0020095 was significantly increased in four CC cell lines, HT29, SW480, SW620, and HCT116, compared to the healthy human colon cell line NCM460 (Figure 1J). We further verified its upregulation in 20 CC tissue samples compared to matched normal samples through qRT-PCR (Figure 1K). In addition, we revealed that circ_0020095 expression was higher in metastatic CC tissues than in non-metastatic tissues (Figure 1L). These results suggested that circ_0020095 was increased in CC, indicating that it might be involved in the pathogenesis of CC.

### Silencing of Circ_0020095 Suppressed CC Cell Growth *in vitro*

**FIGURE 2:**
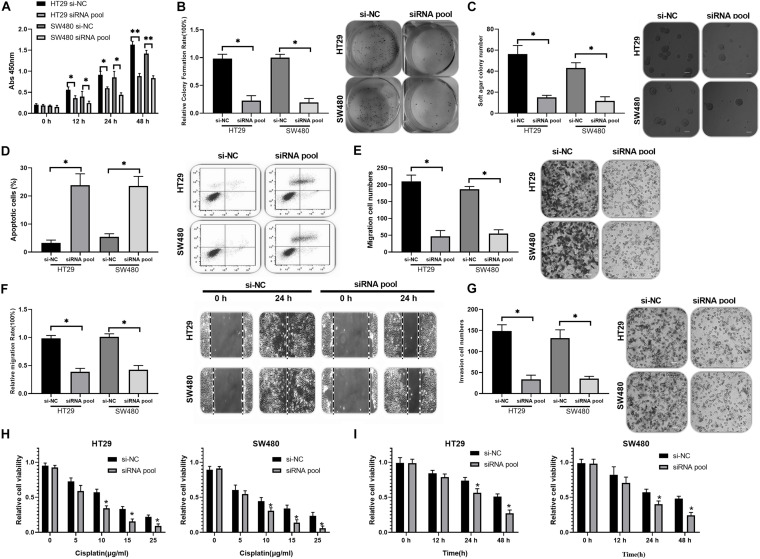
Silencing of circ_0020095 suppressed CC cell growth *in vitro*. **(A)** The viability of HT29 and SW480 cells was determined after 0, 24, and 48 h of si-NC and siRNA pool transfection. After 24 h of si-NC and siRNA pool transfection, HT29 and SW480 cells were subjected to **(B)** colony formation, **(C)** soft agar and **(D)** flow cytometry assays. **(E)** Transwell and **(F)** wound-healing experiments were performed to detect the migratory ability of HT29 and SW480 cells transfected with si-NC or the siRNA pool. **(G)** The impact of circ_0020095 knockdown on the invasive capacity of HT29 and SW480 cells was estimated. **(H)** MTT was used to assess the viability of circ_0020095-silenced HT29 and SW480 cells treated with different concentrations of cisplatin (0, 5, 10, 15, and 25 μg/ml, 24 h). **(I)** After 0, 12, 24, and 48 h of cisplatin (5 μg/ml) treatment, the viability of circ_0020095-silenced HT29 and SW480 cells treated with cisplatin was detected. *P < 0.05, **P < 0.01, ***P < 0.001.

To investigate the functions of circ_0020095 in CC, siRNA#1 and siRNA#2 were stably transfected into HT29 and SW480 cells, followed by analysis of cell proliferation, apoptosis, migration, and invasion. The CCK-8 assay indicated that circ_0020095 knockdown remarkably reduced the viability of HT29 and SW480 cells (Figure 2A). The results from the colony formation assay showed a significant downregulation of the colony formation rate of the siRNA pool groups compared to the si-NC groups (Figure 2B). The soft agar colony formation assay also revealed an inhibition of colony numbers in siRNA pool-transfected HT29 and SW480 cells (Figure 2C). On the other hand, circ_0020095 silencing was demonstrated to cause a dramatic increase in the apoptosis of HT29 and SW480 cells (Figure 2D). The effects of circ_0020095 knockdown on cell migratory capacity were assessed through transwell and wound-healing experiments in HT29 and SW480 cells. The results showed that the migratory capacity of HT29 and SW480 cells was dramatically suppressed in the siRNA pool group compared to the si-NC group (Figures 2E,F). Moreover, by using a Matrigel-coated transwell chamber, we demonstrated that circ_0020095 knockdown resulted in a significant decrease in the number of invasive HT29 and SW480 cells (Figure 2G). To explore the role of circ_0020095 in the chemoresistance of CC cells, HT29 and SW480 cells were treated with different concentrations of cisplatin followed by viability examination using the CCK-8 assay. In the presence of different concentrations of cisplatin (0, 5, 10, 15, and 25 μg/ml), the viability of HT29 and SW480 cells that were transfected with the siRNA pool was markedly suppressed compared to that of cells transfected with si-NC (Figure 2H). Moreover, we found that the viability of HT29 and SW480 cells treated with the siRNA pool was significantly attenuated after 24 and 48 h of cisplatin treatment (5 μg/ml) (Figure 2I). Overall, silencing of circ_0020095 was demonstrated to inhibit CC cell growth *in vitro*.

### Circ_0020095 Acted as a Sponge of miR-487a-3p

**FIGURE 3:**
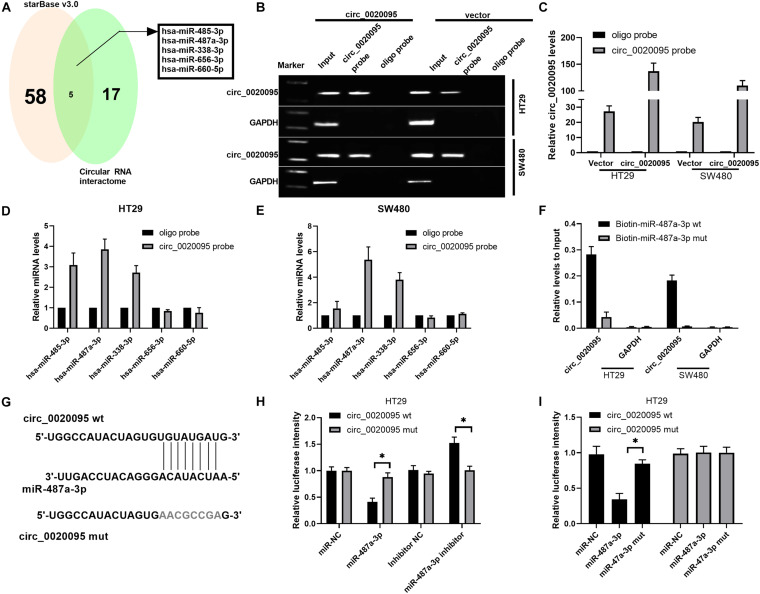
Circ_0020095 acted as a sponge of miR-487a-3p. **(A)** StarBase (v3.0) and Circular RNA interactome were used to predict the target miRNAs of circ_0020095. **(B,C)** Lysates of vector- or circ_0020095-transfected HT29 and SW480 cells were subjected to RNA pulldown using a specific circ_0020095 probe, followed by RT-PCR and qRT-PCR. **(D,E)** Relative expression levels of miR-485-3p, miR-487a-3p, miR-338-3p, miR-656-3p, and miR-660-5p in HT29 and SW480 lysates pulled down by the circ_0020095 probe or oligo probe. **(F)** Circ_0020095 expression in HT29 and SW480 cells captured by biotin-miR-487a-3p wt or biotin-miR-487a-3p mut. **(G)** Schematic of circ_0020095-WT and circ_0020095-MUT luciferase reporter vectors. **(H,I)** A luciferase reporter assay was performed in HT29 and SW480 cells to validate the interaction between circ_0020095 and miR-487a-3p. *P < 0.05, **P < 0.01, ***P < 0.001.

To determine whether circ_0020095 could sponge miRNAs in CC cells, we selected five candidate miRNAs by overlapping the results of the predicted miRNA binding sites in the circ_0020095 sequence by StarBase and Circular RNA interactome (Figure 3A). We then examined whether the identified miRNAs could directly bind circ_0020095. A specific biotin-labeled circ_0020095 probe was designed and used to pull down circ_0020095 in HT29 and SW480 cells. The pulldown efficiency was dramatically increased in HT29 and SW480 cells with stable circ_0020095 overexpression (Figures 3B,C). After pulldown, miRNAs were isolated, and the five candidate miRNAs were analyzed via qRT-PCR. The results indicated that in both HT29 and SW480 cells, miR-487a-3p, and miR-338-3p were abundantly pulled down by the circ_0020095 probe (Figures 3D,E). On the other hand, we found a significant upregulation of circ_0020095 in HT29 and SW480 cells treated with biotin-labeled miR-487a-3p wt compared to those cells treated with biotin-labeled miR-487a-3p mut (Figure 3F). We further performed a luciferase reporter assay to determine whether miR-487a-3p directly interacts with circ_0020095 (Figure 3G). HT29 and SW480 cells cotransfected with miR-487a-3p mimics and a circ_0020095 wt reporter plasmid exhibited reduced luciferase activity (Figures 3H,I). These findings suggested that circ_0020095 acted as a sponge of miR-487a-3p in CC cells.

### Transfection of miR-487a-3p Suppressed CC Cell Growth *in vitro*

**FIGURE 4:**
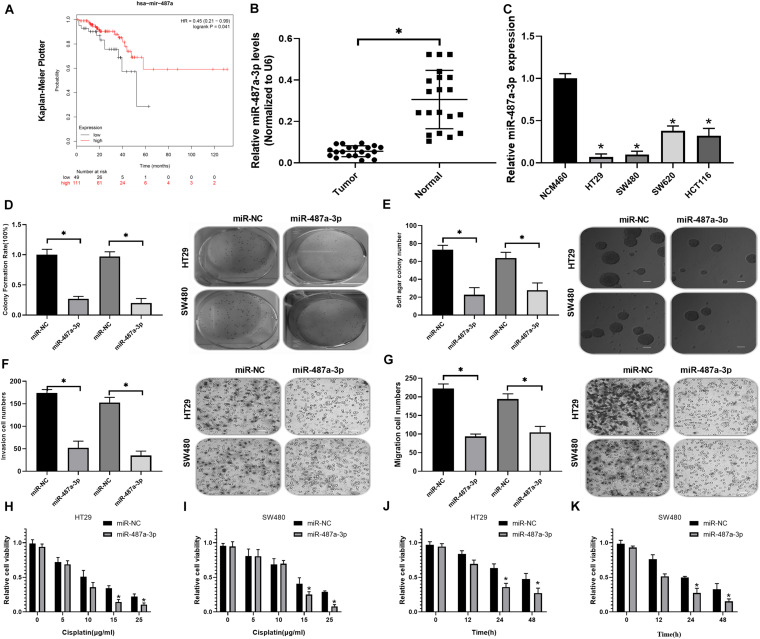
Transfection of miR-487a-3p suppressed CC cell growth *in vitro*. **(A)** Kaplan-Meier analysis of the prognosis of CC patients with high or low miR-487a-3p expression. **(B)** Relative expression level of miR-487a-3p in CC tissue samples and matched normal samples. **(C)** Relative expression level of miR-487a-3p in CC cell lines HT29, SW480, SW620, and HCT116. NCM460 was used as a control. After 24 h of miR-NC and miR-487a-3p mimic transfection, HT29 and SW480 cells were subjected to analysis of proliferation, invasion and migration through **(D)** colony formation, **(E)** soft agar **(F,G)** and transwell assays. (**H**,**I**) The viability of HT29 and SW480 cells transfected with miR-487a-3p or miR-NC was detected in the presence of various concentrations of cisplatin (0, 5, 10, 15, and 25 μg/ml). **(J,K)** After 0, 12, 24, and 48 h of cisplatin (5 μg/ml) treatment, the viability of HT29 and SW480 cells treated with miR-487a-3p or miR-NC was detected. *P < 0.05, **P < 0.01, ***P < 0.001.

Kaplan-Meier analysis indicated that CC patients with low miR-487a-3p expression exhibited a worse prognosis (Figure 4A). By analyzing the expression level of miR-487a-3p with qRT-PCR, we revealed that miR-487a-3p was significantly decreased in both CC tissues and cell lines (HT29, SW480, SW620, and HCT116) compared to normal tissues and the NCM460 cell line, respectively (Figures 4B,C). Next, we estimated the effects of miR-487a-3p overexpression on cell proliferation, invasion and migration. In the colony formation assay, we observed that miR-487a-3p transfection resulted in a significant suppression of the colony formation rate of HT29 and SW480 cells (Figure 4D). Similarly, miR-487a-3p transfection also caused a decrease in the soft agar colony number of HT29 and SW480 cells (Figure 4E). In the transwell assay, we found that miR-487a-3p transfection led to a dramatic suppression of the invasive and migratory abilities of HT29 and SW480 cells compared to miR-NC transfection (Figures 4F,G). In addition, we also investigated the role of miR-487a-3p in the cisplatin resistance of CC cells. The results indicated that miR-487a-3p transfection significantly enhanced the sensitivity of HT29 and SW480 cells to 15 and 25 μg/ml cisplatin compared to the miR-NC transfection (Figures 4H,I). Moreover, the viability of HT29 and SW480 cells was found to be significantly attenuated by overexpressing miR-487a-3p after 24 and 48 h of 5 μg/ml cisplatin treatment (Figures 4J,K). These results demonstrated that miR-487a-3p overexpression remarkably suppressed CC cell growth *in vitro*.

### miR-487a-3p Repressed SOX9 Expression by Directly Binding Its 3′UTR

**FIGURE 5:**
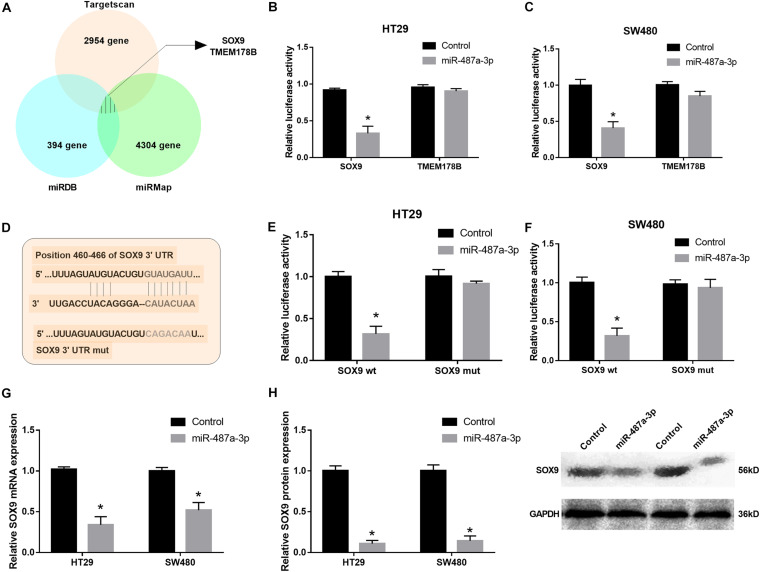
miR-487a-3p repressed SOX9 expression by targeting it. **(A)** TargetScan, miRDB, and miRMap were used to identify the target genes of miR-487a-3p. **(B,C)** The interaction between miR-487a-3p and SOX9 or TMEM178B was determined by dual-luciferase reporter assay. **(D)** Schematic of SOX9-WT and SOX9-MUT luciferase reporter vectors. **(E,F)** Luciferase reporter validation in HT29 and SW480 cells cotransfected with miR-487a-3p mimics and the SOX9-WT or SOX9-MUT plasmid. **(G)** Relative mRNA and **(H)** protein expression of SOX9 in HT29 and SW480 cells transfected with miR-NC or miR-487a-3p. *P < 0.05, **P < 0.01, ***P < 0.001.

To determine the target gene of miR-487a-3p in CC cells, we selected two candidate genes, SOX9 and TMEM178B, by overlapping the results of TargetScan, miRDB and miRMap analyses (Figure 5A). To confirm whether miR-487a-3p physically interacts with these two candidate genes, dual-luciferase reporter assays were carried out in HT29 and SW480 cells. The results indicated that the luciferase activity of CC cells was dramatically attenuated after cotransfection with miR-487a-3p and the SOX9 plasmid but not with the TMEM178B plasmid (Figures 5B,C). We further validated the interaction between miR-487a-3p and SOX9 using SOX9 wt and SOX9 mut reporter plasmids (Figure 5D). The luciferase activity of HT29 and SW480 cells driven by SOX9 wt was obviously reduced by miR-487a-3p, while the luciferase activity driven by SOX9 mut was not affected by miR-487a-3p transfection (Figures 5E,F). In addition, a significant downregulation of SOX9 mRNA and protein levels was observed in miR-487a-3p-overexpressing HT29 and SW480 cells (Figures 5G,H). These findings suggested that miR-487a-3p could suppress the expression of SOX9 in HT29 and SW480 cells by directly binding to SOX9.

### Circ_0020095 Promotes Oncogenesis of Colon Cancer by Sponging Multiple miRNAs

**FIGURE 6:**
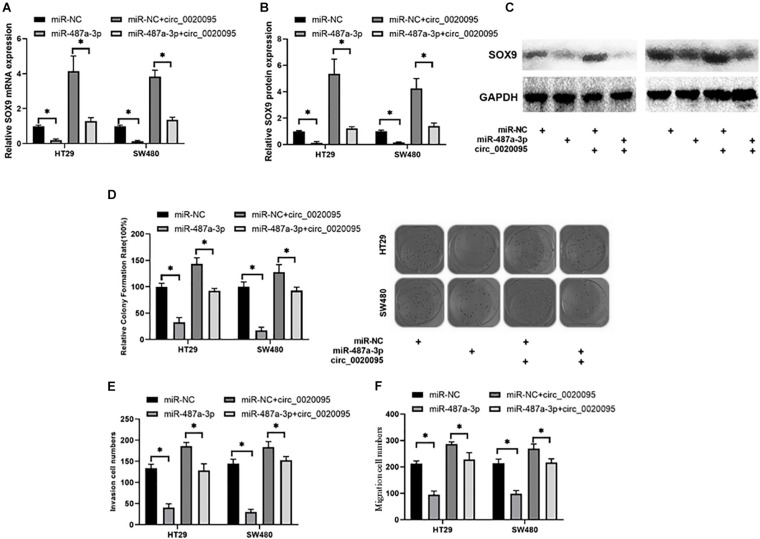
Circ_0020095 abolished the suppressive effects of miR-487a-3p on CC. **(A)** Relative mRNA and **(B,C)** protein expression levels of SOX9 in HT29 and SW480 cells were measured after 24 h of transfection with miR-487a-3p alone or together with circ_0020095. HT29 and SW480 cells were transfected with miR-NC, miR-487a-3p, miR-NC+circ_0020095 or miR-487a-3p+circ_0020095. After 24 h of transfection, these groups of cells were subjected to **(D)** cell proliferation, **(E)** invasion, and **(F)** migration assays. *P < 0.05, **P < 0.01, ***P < 0.001.

Due to the association of circ_0020095 and miR-487a-3p in CC, we next investigated whether circ_0020095 could abolish the inhibitory effects of miR-487a-3p on SOX9 expression and CC cell proliferation, invasion and migration. The results from qRT-PCR and western blot experiments indicated that miR-487a-3p-induced downregulation of SOX9 mRNA and protein was abolished by the cotransfection of miR-487a-3p and circ_0020095 in HT29 and SW480 cells (Figures 6A–C). In the colony formation assay, we observed that ectopic expression of circ_0020095 in HT29 and SW480 cells restored the cell proliferation activity suppressed by miR-487a-3p (Figure 6D). Moreover, by using a transwell assay, we found that circ_0020095 transfection in HT29 and SW480 cells could restore the cell invasion and migration abilities, which were suppressed by overexpression of miR-487a-3p (Figures 6E,F). These results indicated that circ_0020095 could regulate CC cell proliferation, migration and invasion by sponging miR-487a-3p. To further confirm that circ_0020095 serves as a modulator of CC tumorigenesis by sponging multiple miRNAs, we also showed that circ_0020095 regulated Met expression through miR-338-3p (Supplementary Figure 1). These data indicated that circ_0020095 promotes tumorigenesis of CC by sponging multiple miRNAs.

### Silencing of Circ_0020095 Suppressed CC Tumor Growth *in vivo*

**FIGURE 7:**
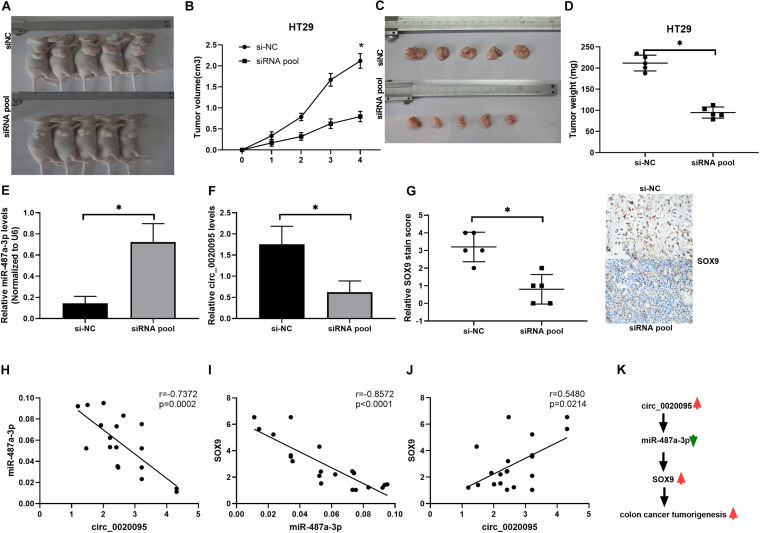
Silencing of circ_0020095 suppressed CC tumor growth *in vivo*. HT29 cells stably transfected with si-NC or the siRNA pool were transferred into BALB/c nude mice. **(A)** Representative images of xenograft tumors. Tumor volume **(B,C)** and **(D)** weight were measured in the si-NC and siRNA pool groups. **(E,F)** Relative expression levels of miR-487a-3p and circ_0020095 were detected in the xenograft tumors collected from the si-NC and siRNA pool groups by qRT-PCR. **(G)** Tumors were collected and cut into 35 μm slices followed by staining for SOX9. Correlations between the expression of **(H)** circ_0020095 and miR-487a-3p, **(I)** miR-487a-3p and SOX9, and **(J)** SOX9 and circ_0020095 were analyzed in the tumor samples. **(K)** Schematic of the potential molecular mechanism of circ_0020095 in the tumorigenesis of CC. *P < 0.05, **P < 0.01, ***P < 0.001.

To further examine whether circ_0020095 silencing could inhibit CC tumor growth *in vivo*, HT29 cells stably transfected with an siRNA pool or si-NC were inoculated into nude mice. Xenograft tumors were examined 4 weeks after inoculation. The results showed that the tumor volumes and weights in the siRNA pool-transfected group were dramatically decreased than those in the si-NC group (Figures 7A–D). The expression levels of miR-487a-3p and circ_0020095 were determined in the xenograft tumors collected from the siRNA pool and si-NC groups. Compared to that in the si-NC group, the expression level of miR-487a-3p was remarkably upregulated, while circ_0020095 was significantly downregulated, in the siRNA pool group (Figures 7E,F). Moreover, by using IHC analysis, we found that the staining intensity of SOX9 was obviously weaker in the tumor sections of the siRNA pool group than in those of the si-NC group (Figure 7G). In addition, in xenograft tumors, a negative correlation was observed between the expression levels of miR-487a-3p and circ_0020095 or SOX9 (Figures 7H,I), while a positive correlation was revealed between the expression levels of circ_0020095 and SOX9 (Figures 7J,K). These results demonstrated that silencing circ_0020095 suppressed CC tumor growth *in vivo*.

## Discussion

Hsa_circ_0020095 was abundantly expressed in CC tissues and CC cell lines. Via *in vitro* and *in vivo* experiments, we have found that circ_0020095 promotes the proliferation, migration and invasion activities in CC, while the circ_0020095/miR-487a-3p sponge structure alleviates these activities in CC. Meanwhile, the expression of SOX9 was in line with the effects by circ_0020095 and circ_0020095/miR-487a-3p sponge structure.

CircRNAs widely exist in the eukaryotic transcriptome and are involved in the modulation of gene expression; therefore, circRNAs have been a research hotspot in recent decades. Circ_0020095 is encoded by the ATRNL1 gene, which might play a role in the melanocortin cascade that modulates energy homeostasis (Stark et al., 2010). The involvement of circRNAs in the initiation and development of human cancers has been well demonstrated by *in vitro* and *in vivo* evidence (Meng et al., 2017). Whether circ_0020095 can affect the progression of CC remains undetermined. Therefore, investigating circRNA regulatory mechanisms to cancer progression will provide insights into future tumor prevention and therapy strategies.

Many circRNAs have been reported in CC thus far. For example, circRNA CCDC66 is identified to be strongly increased in the colon and associated with a poor prognosis. Gain- and loss-of-function studies show that it could promote CC cell growth and metastasis (Hsiao et al., 2017). Similarly, circ_000984 is strongly increased in CC, and its knockdown results in suppression of CC cell proliferation, migration and invasion *in vivo* and *in vitro* (Xu et al., 2017). Moreover, hsa_circ_0136666 is highly expressed in CRC and the silence of hsa_circ_0136666 regulates the proliferation and migration of colorectal cancer (CRC) cells by sponging miR-136, thus modulating the expression of SH2B adaptor protein 1 (SH2B1) (Jin et al., 2019). In accordance with our findings, circ_0020095 was identified to be strongly upregulated in CC tissues and cell lines (HT29 and SW480). After silencing of circ_0020095 with siRNA, CC cell proliferation, migration and invasion were significantly reduced, while the apoptosis was dramatically increased in HT29 and SW480 cells. Similarly, the silence of hsa_circ_0007534 by siRNA remarkably reduces the proliferation and increases apoptosis of CRC cells (Zhang et al., 2018). Moreover, we have found that the cisplatin resistance of circ_0020095 was in a dose- and time-dependent manner (Figures 2H,I). Accumulating evidence has proved that the dysregulation of circRNAs is correlated with drug resistance in various tumors (Hua et al., 2019). Taking CRC as an example, the upregulated hsa_circ_0000504 promotes 5-fluorouracil (5-FU) resistance by modulating the circRNA/miR-485-5p/STAT3/AKT3/BCL2 signaling pathway (Xiong et al., 2017). Taken together, these findings substantiated that circ_0020095 exerted oncogenic effects on CC.

In terms of the mechanism, we chose miR-487a-3p as a potential target miRNA of circ_0020095 by bioinformatics prediction analysis. By searching the literature, we found that miR-487a-3p was only reported in a few studies. In 2018, miR-487a-3p is identified as a lymph node metastasis-associated miRNA in The Cancer Genome Atlas lung adenocarcinoma patient cohort, and a high level of miR-487a-3p was demonstrated to be related to a worse prognosis (Gonzalez-Vallinas et al., 2018). In the same year, miR-487a-3p is revealed to be overexpressed in type 1 diabetes (T1D) by using microarray analysis in the peripheral blood samples of T1D pediatric patients, however, a functional study is not performed (Zurawek et al., 2018). Recently, miR-487a-3p is found to function as a novel tumor repressor in prostate cancer by targeting cyclin D1 (CCND1) (Wang et al., 2020). However, a low level of miR-487a-3p is linked to a high metastasis rate and poor prognosis by targeting SMAD7 in pancreatic cancer tissues (Zhou et al., 2020). To the best of our knowledge, the role of miR-487a-3p in CC has not been reported until now. Through a dual-luciferase reporter assay, we confirmed that in CC cells, circ_0020095 directly binds to miR-487a-3p as a sponge structure which directly targets the 3′-UTR of SOX9 by comparing wt with mut of SOX9 reporter plasmids. Similarly, circRNA-ACAP2 could regulate Tiam1 expression by abolishing the suppressive effect of miR-21-5p on Tiam1 expression by sponging with a miRNA in SW480 cells (He et al., 2018). Moreover, overexpression of miR-487a-3p could suppress CC cell growth. This is the first evidence that miR-487a-3p acts as a tumor suppressor in CC.

SOX9 is a high mobility group box transcription factor that has been shown to be increased in multiple human tissues and acts as an oncogenic agent in tumor progression (Huang and Guo, 2017). A relationship between SOX9 and CC cell growth and development has been observed by a number of studies (Marcker Espersen et al., 2016; Prevostel and Blache, 2017). For this relationship, Shen et al. have found that SOX9 was overexpressed in colon cancer. Knockdown of SOX9 expression results in reduced invasiveness and metastasis of colon cancer cells and inhibits the tumor growth and peritoneal metastasis in nude mice by inhibiting the S100P/RAGE/ERK/EMT signaling pathway (Shen et al., 2015). Similarly, SOX9 regulates migration and invasion in SW480 and SW620 cells and triggers the transition between epithelial and mesenchymal states (Carrasco-Garcia et al., 2016). The expression level of SOX9 could be used to predict relapse in CC patients with stage II disease; a high level predicted a low risk of relapse, and a low level predicted a high risk of relapse (Marcker Espersen et al., 2016). Moreover, miR-133b directly targets the protein level of WAVE2/Sox9/c-Met in clinical samples (Wang et al., 2018). Based on these findings, the potential mechanism of SOX9/c-Met may play an important role during relapse in CC patients. Similarly, we have found miR-487a-3p regulated the protein expression of SOX9 in our experiments. Taken together, circ_0020095 not only indirectly regulated the expression of SOX9 through miR-487a-3p but also reversed the inhibitory effects of miR-487a-3p on CC cells. In our future research, we aim to investigate the mutual effects of SOX-9 and C-Met on CC patients with different stages.

There are some limitations to our study. Firstly, Considering the low expression of miR-487a-3p in both CC tissues and cell lines (HT29, SW480, SW620, and HCT116) compared to normal tissues and the NCM460 cell line, there is another possibility that Sox9 is directly regulated by circ_0020095, or through other targets. However, we have confirmed the finding that circ_0020095 could downregulate the mRNA and protein expression of Sox9 by sponging miR-487a-3p. Secondly, we have only randomly chosen 6 paired CC tumor tissues and adjacent normal tissues to test the expression of 11 circular RNAs. Although the number size is small, we have proved the increased circ_0020095 in tissues (Figure 1K) and cell lines through semiquantitative PCR, FISH assay and qRT-PCR which was consistent with the result in Figure 1B.

## Conclusion

Our results showed that the circ_0020095/miR-487a-3p/SOX9 axis plays a critical role in the progression of CC cells, not only improving our understanding of the tumorigenesis of CC but also offering a novel therapeutic strategy for CC patients.

## Data Availability Statement

The raw data supporting the conclusions of this article will be made available by the authors, without undue reservation.

## Ethics Statement

The studies involving human participants were reviewed and approved by the Research Ethics Committee of Shandong Cancer Hospital and Institute, Shandong First Medical University and Shandong Academy of Medical Sciences. The patients/participants provided their written informed consent to participate in this study. The animal study was reviewed and approved by the Research Ethics Committee of Shandong Cancer Hospital and Institute, Shandong First Medical University and Shandong Academy of Medical Sciences.

## Author Contributions

DM and YS conceived and designed the experiments. DM, ZC, JS, YG, and XL performed the data analysis and interpretation. ZL performed the bioinformatics analysis. YS contributed to clinical materials. DM and ZC were involved in the manuscript preparation. ZL, JS, YG, and XL were responsible for the writing revision and modifications of figures. All authors read and approved the final manuscript.

## Conflict of Interest

The authors declare that the research was conducted in the absence of any commercial or financial relationships that could be construed as a potential conflict of interest.

## Supplementary Material

Supplementary Figure 1 Circ_0020095 functions as an efficient miR-338-3p sponge and regulates Met expression in colon cancer cells. **(A)** Schematic of the circ_0020095-WT and circ_0020095-MUT luciferase reporter vectors. **(B,C)** A luciferase reporter assay was performed in HT29 and SW480 cells to validate the interaction between circ_0020095 and miR-338-3p. **(D)** The protein expression of Met in HT29 and SW480 cells was measured after 24 h of transfection with miR-338-3p alone or together with circ_0020095. Data in **(B,C)** are the mean ± SEM. of three experiments, ^∗^*P* < 0.05, Student’s *t*-test.

## Abbreviations

CC: colon cancer
CCND1: cyclin D1
CircRNAs: circular RNAs
CRC: colorectal cancer
miRNAs: microRNAs
MTT: 3-[4,5-dimethylthiazol-2-yl]-2,5-diphenyl tetrazolium bromide
qRT-PCR: quantitative real-time PCR
SH2B1: SH2B adaptor protein 1
SOX9: SRY-box transcription factor 9
T1D: type 1 diabetes
5-FU: 5-fluorouracil.
