# Supplementary figures and images for: Hsa_circ_0020095 Promotes Oncogenesis and Cisplatin Resistance in Colon Cancer by Sponging miR-487a-3p and Modulating SOX9
Source: Front Cell Dev Biol. 2021 Jan 15;8:604869. doi: 10.3389/fcell.2020.604869 (PMC7844065; doi:10.3389/fcell.2020.604869)

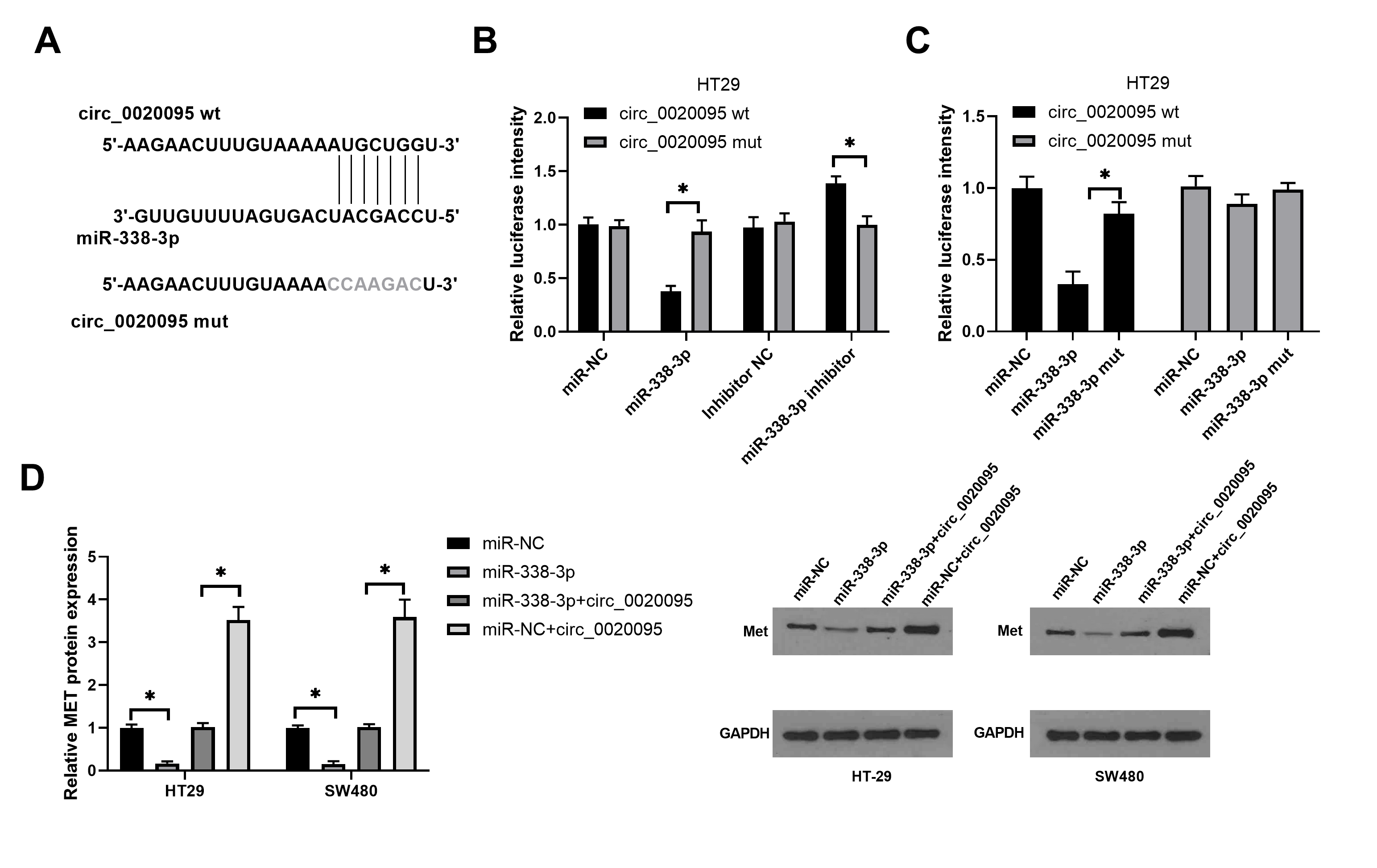

Supplement: Supplementary Figure 1 — Circ_0020095 functions as an efficient miR-338-3p sponge and regulates Met expression in colon cancer cells. (A) Schematic of the circ_0020095-WT and circ_0020095-MUT luciferase reporter vectors. (B,C) A luciferase reporter assay was performed in HT29 and SW480 cells to validate the interaction between circ_0020095 and miR-338-3p. (D) The protein expression of Met in HT29 and SW480 cells was measured after 24 h of transfection with miR-338-3p alone or together with circ_0020095. Data in (B,C) are the mean ± SEM. of three experiments, ∗P < 0.05, Student’s t-test. [file Image_1.TIF]
